# Carbohydrate and protein adaptation in low-protein diets for livestock and poultry: a nutritional revolution from static balance to dynamic synergy

**DOI:** 10.1186/s40104-026-01507-7

**Published:** 2026-10-03

**Authors:** Shuang Cai, Jiayu Tu, Tongtong Yang, Shiyan Qiao, Xiangfang Zeng

**Affiliations:** 1 State Key Laboratory of Animal Nutrition and Feeding, College of Animal Science and Technology, China Agricultural University, No. 2 Yuanmingyuan West Road, Haidian District, Beijing, 100193 People’s Republic of China; 2 Frontier Technology Research Institute of China Agricultural University in Shenzhen, Shenzhen, China

**Keywords:** Carbohydrate and protein adaptation, Dynamic nutrition, Intestinal health, Low-protein diet, Metabolic regulation

## Abstract

Low-protein diet is a pivotal strategy for alleviating protein feed shortage and promoting the sustainable development of the livestock industry. However, the utilization efficiency of carbohydrate and protein nutrients in livestock and poultry remains relatively low, and carbohydrate-protein metabolism asynchrony is the core bottleneck limiting their efficient utilization. Carbohydrate and protein adaptation, based on low-protein diets, makes a shift from traditional static energy and nitrogen balance theories. By precisely aligning the digestive release patterns of carbohydrates and proteins, it enables the synergistic and efficient utilization of energy and amino acids, representing a promising concept in the low-protein diet systems. Nevertheless, current evidence supporting carbohydrate and protein adaptation remains relatively limited and largely based on indirect inference. This review summarizes the patterns of digestion and absorption of carbohydrate and protein in livestock and poultry as well as their key regulatory factors, clarifies the core technology and cutting-edge research methodologies for carbohydrate and protein adaptation, and analyzes the mechanisms by which carbohydrate and protein adaptation influences production performance, meat quality, intestinal health, and nutritional metabolism regulation. In addition, this review proposes future research directions, including quantitative and precise regulation, microbiome-targeted modulation, and multi-omics mechanism analysis, providing a theoretical basis for further research and industrial application of carbohydrate and protein adaptation in low-protein diets for livestock and poultry.

## Introduction

**Fig. 1:**
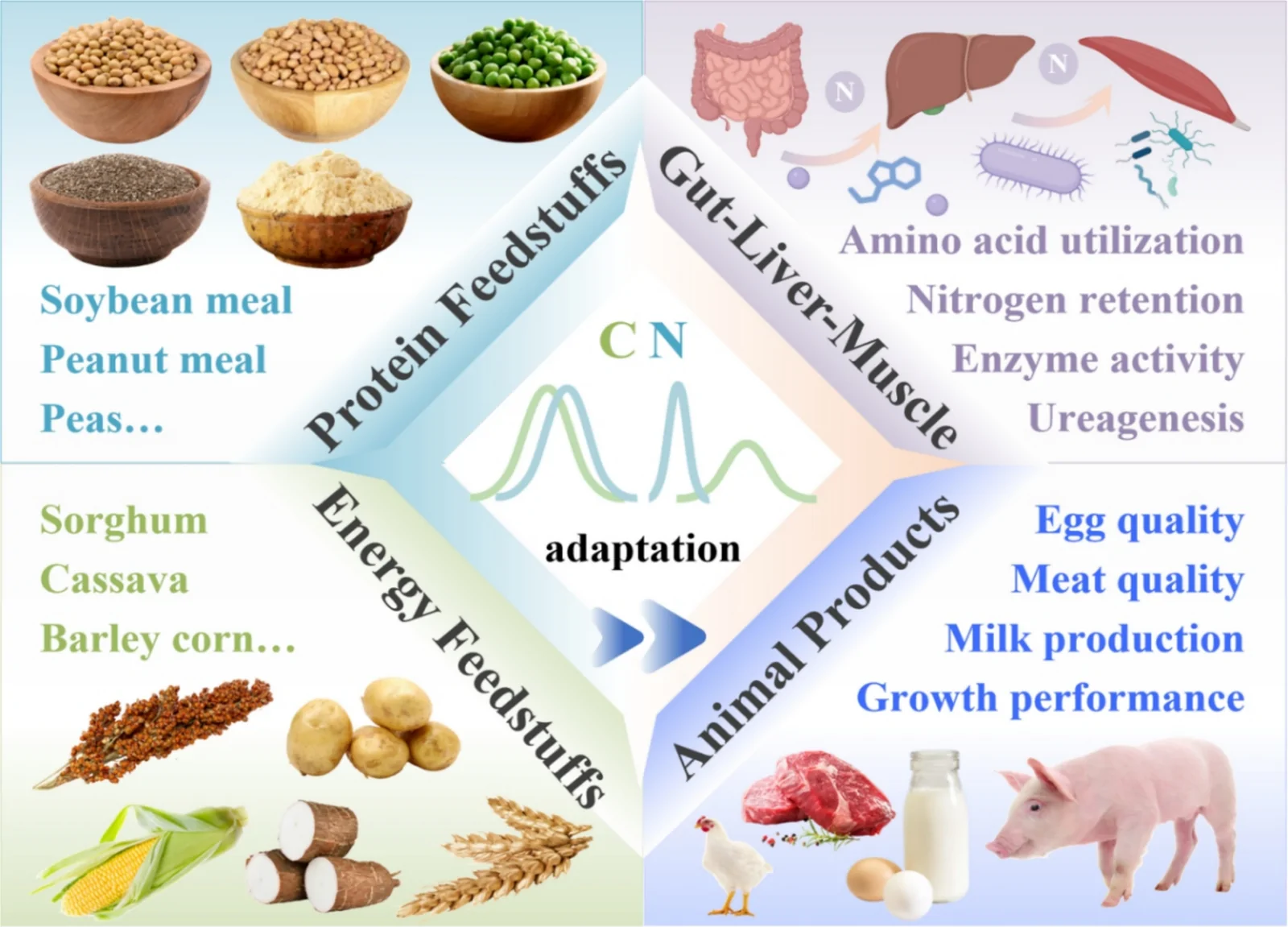
Effects of protein feedstuffs and energy feedstuffs on carbohydrate (C) and protein (N) metabolism and animal products in livestock. The figure was created in BioRender (https://biorender.com/zsvxzrs)

Low-protein diets are formulated based on the actual protein requirements of livestock and poultry, as well as theories of energy and protein balance. Supplemented with appropriate types and amounts of industrially synthesized amino acids, low-protein diets can ensure animal productivity and product quality while maintaining low protein levels, reducing nitrogen emissions, and improving animal intestinal health and the farming environment [1–3]. Many studies have shown that feeding low-protein diets to livestock and poultry can effectively reduce the amount of protein in feed and reduce the nitrogen emission of livestock and poultry, achieving the integration of reductions in nitrogen consumption and emissions with quality improvement and efficiency gains [4, 5]. Feeding low-protein diets is a key technical approach to alleviating the shortage of protein feed resources and ensuring feed grain security. However, the current utilization efficiency of carbohydrate and protein nutrients in livestock and poultry remains generally low. For carbohydrate and protein nutrients, pigs have utilization efficiencies of only 40% and 52%, respectively, while poultry have efficiencies of 35% and 48% [6–8]. In ruminants, the apparent digestibility of nitrogen is even lower, at 25%−35%, resulting in a significant waste of feed resources [9, 10]. The core cause lies in the energy and protein imbalance, yet existing concepts fall short of resolving this bottleneck: energy-protein synchronization focuses on the static matching of total dietary energy and crude protein at the diet level, without addressing the kinetic dynamics of nutrient utilization; nutrient supply kinetics describes the release profile of nutrients but remains a single-nutrient index, failing to address how the kinetics of multiple nutrients interact; dynamic nutrient formulation adjusts dietary supply over days or weeks according to physiological state; and nutrient synchronization, well-established in ruminant nutrition, optimizes microbial protein synthesis by matching carbohydrate and protein degradation in the rumen, but it does not extend to post-ruminal, systemic coordination in monogastric animals. Carbohydrate and protein adaptation addresses this gap by targeting the temporal synchronization, spatial coordination, and functional synergy between carbohydrate and protein digestion, absorption, and metabolism under low-protein conditions (Fig. 1).

Recent conceptual developments in low-protein nutrition have highlighted the importance of carbohydrate-protein adaptation. Huang et al. [11] demonstrated that slow glucose release disrupts carbohydrate and protein synchrony, leading to reduced growth performance and increased nitrogen loss, whereas rapid glucose release enables synchronized carbon and nitrogen supply and improves amino acid digestibility using pig models with ileal cannulas. However, research on carbohydrate and protein adaptation remains in the qualitative exploratory phase, and the theoretical framework and technical solutions for precise regulation have yet to be fully developed. Achieving quantitative alignment of carbohydrates and amino acids release rhythms, elucidation of their underlying molecular mechanisms, and adaptation of unconventional feed ingredients remain significant challenges faced by the livestock industry. We made a thorough literature search in Web of Science, with a limitation for articles written in the English language. Search terms used were carbohydrate-protein adaptation, carbon–nitrogen synchrony, low-protein diet, starch source, protein source, nutrient synchronization. In addition, reference sections of all relevant studies or reviews were manually searched for more information. In this review, we evaluate the patterns of digestion and absorption of carbohydrate and protein nutrients, clarify the core technology and cutting-edge research methodologies for carbohydrate and protein adaptation, thoroughly analyze their impact on livestock and poultry health and the underlying regulatory mechanisms, and outline future research directions in this field. This review aims to provide theoretical support for the precise implementation of low-protein diets and advance the shift in animal nutrition from static balance to dynamic synergy.

## Digestion and absorption characteristics of carbohydrate and protein nutrients and influencing factors

The digestion and absorption characteristics of carbohydrates and proteins constitute the physiological foundation for their adaptation, and are influenced by multiple factors such as structural properties, processing methods, and dietary interactions [12–15]. Elucidating the differences in their digestive and absorptive patterns and identifying key regulatory targets are prerequisites for precise carbohydrate and protein adaptation and represent an innovative entry point for nutritional regulation.

The digestion and absorption of carbohydrate and protein in livestock and poultry are complex physiological processes that are jointly determined by the physical properties, chemical structure, processing technology, antinutritional factors and the dietary component interactions [16, 17]. In terms of particle size, reducing particle size increases the specific surface area for enzymatic contact and improves starch digestibility, but the optimal size varies by species: monogastric animals prefer finer particles (500–700 μm for pigs, 800–1,000 μm for poultry), while excessive fineness in ruminants increases the risk of acidosis [18–22]. Regarding structural composition, A-type crystal starch is more difficult to hydrolyze than B-type starch; starches with a lower amylose/amylopectin ratio are digested faster [23, 24]. The compact spatial conformation of proteins (e.g., the disulfide bond network of prolamins) hinders enzymatic hydrolysis, whereas moderate denaturation can expose cleavage sites [25–28]. In terms of processing technology, gelatinization of starch and denaturation of proteins improve digestibility, but excessive heating triggers the Maillard reaction or protein aggregation [29–32]. Enzymatic pretreatment hydrolyzes proteins into small peptides, which can bypass gastric digestion and be directly absorbed in the small intestine [33]. Among antinutritional factors, protease inhibitors, lectins, tannins, and phytic acid inhibit enzyme activity, damage intestinal villi, or form insoluble complexes, leading to a 30%−50% increase in endogenous nitrogen loss [34–37]. Regarding the interactive effects of dietary components, energy deficiency forces amino acids to be used for gluconeogenesis, while excess non-starch polysaccharides increase digesta viscosity and impede enzyme diffusion [38–40]. Zinc and manganese are indispensable as cofactors for peptidases [41, 42]. Phytase not only releases phosphorus but also hydrolyzes phytate-protein complexes, achieving "one enzyme, multiple effects" for the simultaneous improvement of carbon and nitrogen digestion [43]. In summary, the synchrony of carbon and nitrogen digestion is the result of synergistic regulation by multiple physical, chemical, processing, and nutritional factors.

## Technical core and implementation pathways for carbohydrate and protein adaptation in low-protein diets

### Release characteristics of carbohydrates and amino acids

**Fig. 2:**
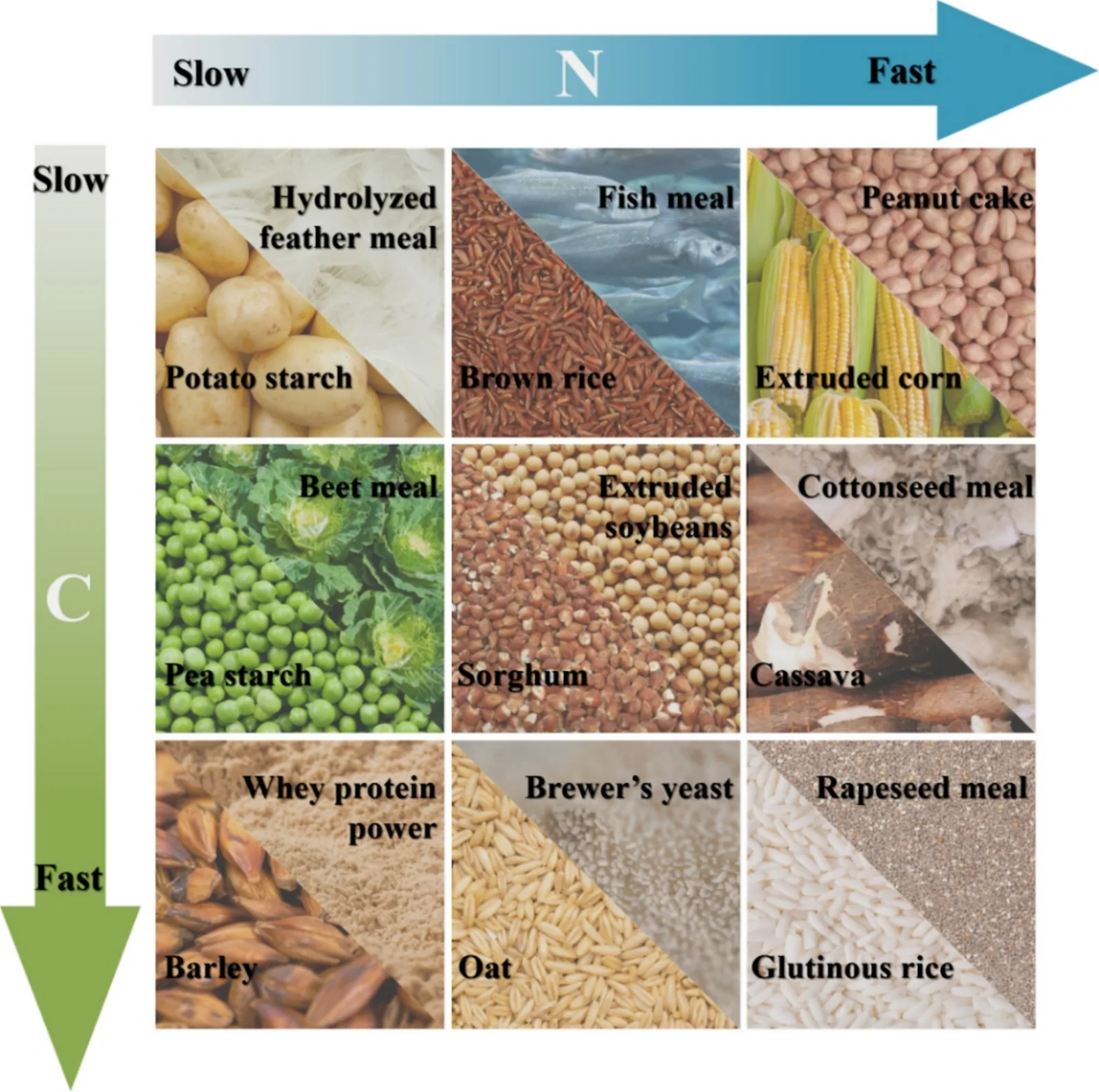
The release rates of carbohydrates and proteins from different feed ingredients along the digestive tract. The figure was created in BioRender (https://BioRender.com/7vx5fcb)

Determining the release characteristics of carbohydrates and amino acids from feedstuffs is crucial for the precise selection and combination of carbohydrate and protein feedstuffs and for the construction of a diet with a reasonable amylose-amylopectin ratio and matched glucose and amino acids release. Taking the classic corn-soybean meal diet as an example, corn, as the main carbohydrate source, has a high proportion of starch and can continuously and stably provide glucose to meet the immediate and sustained demand for energy in pigs [44]. As a high-quality plant-based protein source, soybean meal is not only rich in essential amino acids, such as lysine and tryptophan, but can also be easily and fully digested in the proximal small intestine to release small peptides and amino acids, resulting in a high degree of synchronization with the glucose supplied by the corn. However, because corn distillers dried grains with solubles (DDGS) is characterized by the slow digestion and release of peptides and amino acids, using it to replace a high proportion of soybean meal reduces nitrogen utilization efficiency in growing pigs, leading to a decline in growth performance [45–47]. Because direct determination of glucose and amino acid release kinetics requires extensive in vitro digestion assays and in vivo vascular cannulation, which are technically demanding and costly, direct studies in this field remain limited. On the basis of existing experimental data, this review compared the carbohydrate and amino acid release rates of common energy and protein feedstuffs. Because experimental models, analytical approaches, sampling sites, and sampling times vary among individual studies, we used corn and soybean meal as within-study benchmarks. The starch digestion rate and amino acid release rate of other ingredients were compared to those of corn and soybean meal, respectively, to assess whether their nutrient release was relatively rapid or slow (Fig. 2, Tables 1 and 2).

**Table 1:** Starch digestibility of common energy feed ingredients and diets^a^

Item	Release rate^b^	Starch digestibility	Starch digestibility of corn	Model	Method	Reference
Barley	Slow	93.28%	93.66%	Pig	In vitro digestion^c^	[11]
Barley	Rapid	98.80%	97.40%	Pig	In vitro digestion	[48]
Broken rice	Rapid	97.03%	93.66%	Pig	In vitro digestion	[11]
Brown rice	Rapid	97.66%	93.66%	Pig	In vitro digestion	[11]
Cassava	Slow	70.10%	97.40%	Pig	In vitro digestion	[48]
Cassava	Rapid	95.60%	94.80%	Pig	In vitro digestion	[49]
Corn starch	Slow	87.20%	97.40%	Pig	In vitro digestion	[48]
Extruded corn	Slow	54.00%	97.40%	Pig	In vitro digestion	[48]
Glutinous rice	Rapid	97.00%	65.00%	Poultry	In vitro digestion	[50]
Indica rice	Rapid	81.00%	65.00%	Poultry	In vitro digestion	[50]
Potato starch	Slow	36.10%	97.40%	Pig	In vitro digestion	[48]
Sorghum	Slow	87.40%	97.40%	Pig	In vitro digestion	[48]
Wheat	Rapid	99.40%	97.40%	Pig	In vitro digestion	[48]
Wheat	Slow	72.30%	97.30%	Poultry	In vivo digestion^d^	[51]
Barley	Rapid	95.00%	84.00%	Pig	In vivo digestion	[52]
High-amylose corn starch	Slow	60.00%	84.00%	Pig	In vivo digestion	[52]
Barley	Slow	96.00%	97.00%	Pig	In vivo digestion	[53]
Barley	Slow	94.00%	96.00%	Poultry	In vivo digestion	[53]
Oat	Rapid	98.00%	97.00%	Pig	In vivo digestion	[53]
Oat	Rapid	99.00%	96.00%	Poultry	In vivo digestion	[53]
Glutinous rice	Rapid	92.20%	89.90%	Poultry	In vivo digestion	[50]
Indica rice	Rapid	90.00%	89.90%	Poultry	In vivo digestion	[50]
Barley	Rapid	6.80 mmol/L	6.20 mmol/L	Pig	Portal vein blood collection^e^	[44]
Cassava	Slow	5.90 mmol/L	6.20 mmol/L	Pig	Portal vein blood collection	[44]
Sorghum	Slow	5.50 mmol/L	6.20 mmol/L	Pig	Portal vein blood collection	[44]
Waxy maize starch	Rapid	4.39 mmol/L	4.04 mmol/L	Pig	Jugular vein blood collection^f^	[54]

^a^ References [11, 48] evaluated individual feed ingredients. The other studies evaluated complete diets containing the listed item as the principal starch source

^b^ Rapid and slow classifications were assigned based on numerical comparisons with the corresponding value for corn within the same study

^c^ In vitro digestion simulates the digestion of feed in the stomach and small intestine under controlled laboratory conditions, including temperature, pH, buffer system, digestive enzymes, and incubation time. References [11, 48, 49] used two-stage gastric and intestinal digestion procedures, with pepsin used during the gastric phase and pancreatin, amyloglucosidase, and invertase used during the intestinal phase. References [11, 48] used a 120-min gastric phase followed by a 480-min intestinal phase at 39 °C. Reference [49] used a 30-min gastric phase followed by a 240-min intestinal phase at 39 °C. Reference [50] used a continuous 240-min in vitro Englyst digestion procedure at 37 °C, and the specific digestive phases, enzyme types, and sample particle size were not reported

^d^ In vivo digestion involves feeding experimental diets to animals and estimating the starch digestibility by comparing dietary starch intake with the starch remaining in digesta collected from specific intestinal segments or in excreta. Reference [50] used a 72-h total excreta collection method. References [51–53] represent starch digestibility determined using ileal digesta collected after slaughter. In Reference [51], animals were slaughtered at 35 d of age. In Reference [52], animals were fed the experimental diets for at least 12 d before slaughter. In Reference [53], animals were fed the experimental diets continuously for 10 d before slaughter

^e^ Portal vein blood was collected directly under anesthesia at 4 h after the morning meal on d 35 of the study and the concentration of glucose was detected

^f^ Jugular vein blood was collected using vacuum tubes after a 12-h overnight fast on d 45 of the study and the concentration of glucose was detected

**Table 2:** Amino acid release characteristics of common protein feed ingredients and diets^a^

Item	Release rate^b^	Total amino acid release	Amino acid release of soybean meal	Model	Method	Reference
Cottonseed meal	Rapid	39.09 mmol/g	38.19 mmol/g	Pig	In vitro digestion^c^	[55]
Rapeseed meal	Rapid	47.24 mmol/g	38.19 mmol/g	Pig	In vitro digestion	[55]
Beet meal	Slow	2.76 mmol/g	4.54 mmol/g	Pig	In vitro digestion	[48]
Brewer’s yeast	Rapid	4.64 mmol/g	4.54 mmol/g	Pig	In vitro digestion	[48]
Corn gluten meal	Rapid	6.31 mmol/g	4.54 mmol/g	Pig	In vitro digestion	[48]
Distillers’ grain	Slow	3.50 mmol/g	4.54 mmol/g	Pig	In vitro digestion	[48]
Extruded soybean	Slow	3.88 mmol/g	4.54 mmol/g	Pig	In vitro digestion	[48]
Fermented soybean meal	Slow	3.99 mmol/g	4.54 mmol/g	Pig	In vitro digestion	[48]
Greens cake	Slow	3.78 mmol/g	4.54 mmol/g	Pig	In vitro digestion	[48]
Hydrolyzed feather meal	Slow	2.82 mmol/g	4.54 mmol/g	Pig	In vitro digestion	[48]
Palm kernel meal	Slow	3.64 mmol/g	4.54 mmol/g	Pig	In vitro digestion	[48]
Pea	Rapid	6.80 mmol/g	4.54 mmol/g	Pig	In vitro digestion	[48]
Peanut cake	Rapid	6.20 mmol/g	4.54 mmol/g	Pig	In vitro digestion	[48]
Soybean protein concentrate	Rapid	4.66 mmol/g	4.54 mmol/g	Pig	In vitro digestion	[48]
Sprayed corn germ meal	Slow	3.54 mmol/g	4.54 mmol/g	Pig	In vitro digestion	[48]
Sunflower meal	Rapid	4.89 mmol/g	4.54 mmol/g	Pig	In vitro digestion	[48]
Whey protein powder	Slow	3.59 mmol/g	4.54 mmol/g	Pig	In vitro digestion	[48]
Fish meal	Slow	4.02 mmol/g	4.54 mmol/g	Pig	In vitro digestion	[48]
Corn germ meal	Slow	47.40%	75.20%	Pig	In vitro digestion	[56]
Corn gluten meal	Slow	31.10%	75.20%	Pig	In vitro digestion	[56]
DDGS	Slow	45.10%	75.20%	Pig	In vitro digestion	[56]
Dehulled soybean meal	Slow	73.80%	75.20%	Pig	In vitro digestion	[56]
Fermented soybean meal	Rapid	76.90%	75.20%	Pig	In vitro digestion	[56]
Fish meal	Slow	61.00%	75.20%	Pig	In vitro digestion	[56]
Rapeseed meal	Slow	52.50%	75.20%	Pig	In vitro digestion	[56]
Corn gluten meal	Slow	781.24 mg/L	890.28 mg/L	Pig	In vitro digestion	[57]
Fermented soybean meal	Rapid	940.11 mg/L	890.28 mg/L	Pig	In vitro digestion	[57]
Fish meal	Slow	864.37 mg/L	890.28 mg/L	Pig	In vitro digestion	[57]
Blood meal	Slow	49.00%	91.00%	Poultry	In vitro digestion	[58]
Canola meal	Slow	86.00%	91.00%	Poultry	In vitro digestion	[58]
DDGS	Slow	61.00%	91.00%	Poultry	In vitro digestion	[58]
Corn gluten meal	Slow	80.00%	91.00%	Poultry	In vitro digestion	[58]
Fish meal	Comparable	91.00%	91.00%	Poultry	In vitro digestion	[58]
Hydrolyzed feather meal	Slow	55.00%	91.00%	Poultry	In vitro digestion	[58]
Meat and bone meal	Slow	56.00%	91.00%	Poultry	In vitro digestion	[58]
Dehulled soybean meal	Rapid	18.98 mmol/L	13.9 mmol/L	Poultry	In vitro digestion	[59]
Fermented soybean meal	Rapid	24.07 mmol/L	13.9 mmol/L	Poultry	In vitro digestion	[59]
DDGS	Rapid	2,100 μg/mL	500 μg/mL	Pig	In vivo digestion^d^	[60]
Casein	Rapid	1,000 μg/mL	500 μg/mL	Pig	In vivo digestion	[60]
Blood meal	Slow	43.00%	75.00%	Poultry	In vivo digestion	[61]
Canola meal	Slow	66.00%	75.00%	Poultry	In vivo digestion	[61]
DDGS	Slow	71.00%	75.00%	Poultry	In vivo digestion	[61]
Corn gluten meal	Rapid	84.00%	75.00%	Poultry	In vivo digestion	[61]
Fish meal	Comparable	75.00%	75.00%	Poultry	In vivo digestion	[61]
Hydrolyzed feather meal	Slow	50.00%	75.00%	Poultry	In vivo digestion	[61]
Meat and bone meal	Slow	70.00%	75.00%	Poultry	In vivo digestion	[61]
Blood meal	Rapid	76.00%	74.70%	Poultry	In vivo digestion	[62]
Cold pressed canola meal	Slow	69.70%	74.70%	Poultry	In vivo digestion	[62]
Expeller-pressed canola meal	Slow	68.50%	74.70%	Poultry	In vivo digestion	[62]
Casein	Slow	2,050 μmol/L	3,500 μmol/L	Pig	In vivo vein catheter^e^	[63]

^a^ References [48, 55, 56, 58, 59] evaluated individual protein feed ingredients; References [57, 60, 63] evaluated complete diets formulated with the listed protein sources and References [61, 62] evaluated semi-purified diets formulated with the listed protein sources

^b^ Rapid and slow classifications were assigned based on numerical comparisons with the corresponding value for soybean meal within the same study

^c^ In vitro digestion simulates the digestion of dietary protein in the stomach and small intestine under controlled laboratory conditions, including temperature, pH, buffer system, digestive enzymes, and incubation time. References [48, 55] used two-stage gastric and intestinal digestion procedures, with pepsin used during the 120-min gastric phase and pancreatin, amyloglucosidase, and invertase used during the 480-min intestinal phase at 39 °C. References [56, 57] used two-stage pepsin-pancreatin digestion procedures, consisting of a 4-h gastric phase at 37 °C followed by intestinal digestion at 39 ℃ for 28 h. Reference [58] used a two-stage digestion procedure consisting of a 30-min gastric phase with pepsin followed by a 180-min intestinal phase with pancreatin, both at 41 °C. Reference [59] used a two-step acidic-neutral incubation, consisting of a 60-min acidic phase with pepsin at pH 2.5 and 37 °C followed by a 120-min neutral phase at pH 6.8 and 37 °C; amino acid release was determined in the absence or presence of exogenous multiprotease. The values from Reference [59] presented in Table 2 correspond to the 1,200 U/g multiprotease treatment. Reference [56] represents the extent of total amino acid release from the ingredients. Reference [58] represents protein digestibility

^d^ In vivo digestion involves feeding experimental diets to animals and estimating protein or amino acid digestion from nutrients remaining in digesta collected from specific intestinal segments. In Reference [60], digesta were collected from the ileocecum at 2 h post-feeding on d 28 and total amino acids were detected. In Reference [61, 62], digesta were collected from the distal ileum after euthanasia on d 21 and d 27, respectively and apparent amino acid digestibility was determined

^e^ In vivo vein catheter involves collecting blood from live animals through an indwelling venous catheter. In Reference [63], an indwelling anterior vena cava catheter was inserted via the cephalic vein. Plasma samples were collected at 8 h after feeding on d 5 of the experimental period and total amino acids were detected

### Non starch polysaccharides (NSP) composition and fermentation characteristics

The selection of fiber sources in the diet significantly impacts carbohydrate and protein adaptation. Although fiber materials often contain NSPs, the starch content in oat bran, wheat bran, rice bran, and corn bran is generally higher than that in soybean hull, alfalfa meal, sunflower meal, palm meal, beet pulp, and citrus peel [64]. The glucose released during digestion interferes to some extent with the synchronized release of glucose and amino acids during the digestion of starch and protein. Moreover, there are significant differences in the composition of NSPs among the different types of fiber raw materials. Among them, soybean hull, alfalfa, beet pulp, citrus peel, and oat bran contain higher levels of soluble NSPs, whereas wheat bran and palm meal contain higher levels of insoluble NSPs [65, 66]. The different ratios of soluble and insoluble NSPs in different fiber materials lead to changes in fermentation kinetics, fermentation sites, and short chain fatty acid (SCFA) profiles. In vitro fermentation studies have shown that there are significant differences in the gas production rate and acid production preference of different NSPs, and these differences are further reflected in the forward or backward shift of the fermentation position in vivo, as well as the changes in the relative proportions of SCFAs, such as acetic acid, propionic acid, and butyric acid, which affect the supply of fermentable carbohydrates in the hindgut and intestinal barrier function [67, 68]. Soybean hulls often have high fermentation activity and are biased toward early fermentation, and the SCFA profile is dominated by acetic acid. In contrast, the fermentation rates of corn germ meal and wheat bran after predigestion treatment are relatively low, and these materials can produce a relatively high level of butyric acid within a specific fermentation time window, which is conducive to the maintenance of intestinal health [69, 70]. Similarly, in actual formulas, there is usually more room for the suitable addition of corn germ meal and wheat bran; however, it is necessary to control the amount of fibrous raw materials with high activity such as soybean hull, and the release patterns of carbon and nitrogen sources should be incorporated into the design [71]. Therefore, when these materials are added to the diet, the proportion of polysaccharides should be strictly controlled according to the composition and characteristics of the polysaccharides to avoid adverse effects on growth performance.

## Techniques for exploring carbohydrate and protein adaptation

### Vascular cannulation technique

**Fig. 3:**
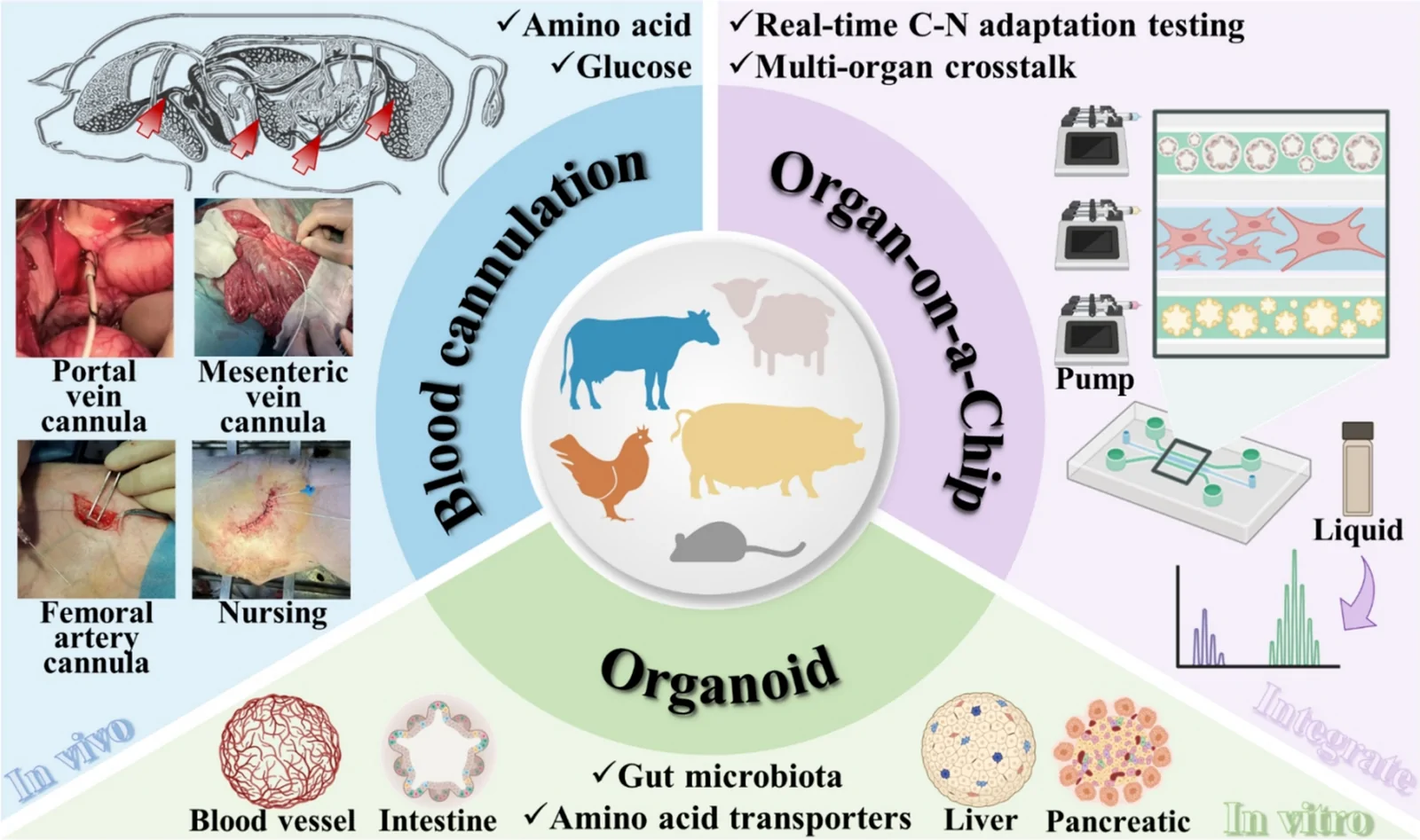
Advanced tools for studying carbohydrate (C) and protein (N) synchronization in livestock. The figure was created in BioRender (https://BioRender.com/c8qo6ja)

Vascular cannulation technique is used to observe dynamic changes of amino acid and glucose concentrations in animals, which involves the placement of a permanent vascular cannula in one or more body sites to enable continuously blood collection or the input of metabolic interventions [72]. Depending on the site of catheter insertion, vascular cannulation can be classified as hepatic vein cannulation, portal vein cannulation, femoral vein cannulation, femoral artery cannulation, mesenteric vein cannulation, or carotid artery cannulation (Fig. 3). In recent years, many studies have applied multisite vascular cannulation to analyze nutrient metabolism patterns in the intestine and liver of animals. Using portal vein cannulation, Lv et al. [73] investigated metabolizable glucose in diets of weaned piglets; Hu et al. [74] fitted catheters into the femoral artery, portal vein and hepatic vein to explore net absorption and liver metabolism of amino acids in sows. Other studies have focused on protein dynamics and metabolism. Zheng et al. [75] implanted catheters in the carotid arterial, jugular venous, femoral arterial and venous catheter to calculate skeletal muscle protein synthesis and degradation in piglets, while Sun et al. [76] studied the effects of adding sodium dichloroacetate to low-protein diets on hepatic amino acid metabolism. However, vascular cannulation is technically demanding. It requires highly experienced surgeons and post-operative care. Cannula fixation and post-operative infection remain critical factors affecting surgical success.

### Organoid

In 2025, the U.S. FDA explicitly announced that it would broadly promote organoids and organs-on-chips to replace animal testing, and the European Union has also formally proposed to gradually phase out animal experiments for chemical safety assessment in favor of organoid-based methods [77]. An organoid is a tissue analog with defined three-dimensional structure, generated by the in vitro culture of somatic cells, adult stem cells (including progenitor cells), or pluripotent stem cells through self-organization, which strongly recapitulates the structure and function of real organs and can be stably cultured for extended periods [78, 79]. Over the past decade, organoid technology has advanced rapidly, leading to successful establishment of organoids for the liver, kidney, lung, breast, fallopian tube, hippocampus, retina, and other organs [80, 81]. These organoids hold great promise for applications in functional tissue induction, disease modeling, drug screening, anti‑inflammatory experiments, and beyond [82].

In recent years, significant progress has been achieved in developing organoid models for livestock and poultry, including pigs, cattle, sheep, horses, and chickens. For the intestinal tract, porcine intestinal organoids have been established and shown to robustly express nutrient transporters, hormones, and digestive enzyme genes, enabling studies on the absorption and metabolic responses to nutrients such as fatty acids [83]. In addition, microfluidic-based porcine small intestinal organoid-on-a-chip models have been successfully developed. Intestinal organoids from dogs, cats, rabbits, pigs, chickens, turkeys, and horses are also actively employed in research, offering versatile platforms for nutritional and physiological studies across species [84–88]. For the liver, a sheep liver organoid model has been reported; however, transcriptomic analysis indicates that these organoids preferentially undergo proliferation and structural remodeling, with relatively downregulated expression of genes associated with mature hepatic metabolic functions [89]. Collectively, while these advances provide valuable tools, standardized and functionally mature organoid models for farm animals remain limited, particularly for metabolic studies requiring full hepatic functionality. Moreover, there is currently no recognized, standardized protocol for culture conditions, passage numbers, and quality control criteria, which vary widely among laboratories, making cross-study comparisons difficult.

### Organ-on-a-chip

Organ-on-a-chip technology integrates organoids onto a microfluidic chip using microtechnology such as lithography, microfabrication, and microfluidics [90, 91]. It simulates the biological microenvironment of internal organs and closely approximates their physiological structure and function. This approach overcomes the limitations of traditional organoid culture, including the inability to establish multiple cell types, difficulty in recapitulating dynamic microenvironments, and restricted nutrient exchange [92]. Organ-on-a-chip technology enables culture, analysis, and detection of organoids from a microscopic perspective, greatly improving research efficiency (Fig. 3).

Multi-organ-on-a-chip systems that integrate gut, liver, and adipose tissue can better model whole-body nutrient metabolism and are highly relevant for carbohydrate-protein adaptation studies. In parallel, many studies reported breakthroughs in wearable electrochemical sensors, for example, the team at MIT achieved continuous, real-time monitoring of metabolites and nutrients in human biofluids [93]. However, most of these advances, whether in organ-on-a-chip or wearable sensors, have been predominantly focused on human applications, especially for tumor disease modeling and drug discovery [94–96]. Consequently, further work is needed to faithfully reproduce the physiological and metabolic functions of animal organs, and more research is required to apply these technologies to animal models.

## Effects of a low-protein diet based on carbohydrate and protein adaptation on the health of livestock and poultry and the underlying regulatory mechanism

### Improvement in growth performance

Numerous studies have shown that the use of feedstuffs with different glucose and amino acid release rates strongly affects the growth performance of livestock and poultry (Table 3). The use of raw materials that slowly release amino acids such as casein is conducive to increasing the daily weight gain and nitrogen use efficiency of pigs [105]. However, if the digestion of protein sources is too slow or the digestibility is insufficient, such as in corn DDGS and soybean protein concentrate, the partial replacement of soybean meal could result in decreased daily gain and increased body protein loss [60]. In addition, the composite mode of starches with different digestibility rates (coexistence of rapid glucose supply and continuous glucose supply) is more conducive to the formation of a stable energy supply, thereby increasing the capture and utilization of nitrogen. Studies on piglets and growing pigs have shown that adding a certain proportion of rapidly digestible starch sources, such as waxy corn, cassava, and barley, helps improve the coordination of glucose and amino acid supply in the intestine, enhances growth performance and nitrogen utilization efficiency, and promotes systemic protein deposition [44, 49, 106]. Rapidly digestible starches typically have a high proportion of amylopectin [107, 108]. Diets rich in amylopectin can increase average daily weight gain, average daily feed intake, nutrient digestibility, and energy deposition in growing pigs, significantly promoting growth performance [109]. Moreover, timely energy support enhances amino acid digestibility and absorption, thereby increasing the net flux of amino acids from the intestine to the portal vein and subsequently to hindlimb muscle [44, 110]. Conversely, slow-release glucose sources are more likely to disrupt carbohydrate and protein synchronization, reduce amino acid digestibility and growth performance, and increase nitrogen loss, indicating that if slowly digestible carbohydrate sources are used, the quality of the protein source and the supply of amino acids must be optimized accordingly to avoid creating new imbalances in nutrient supply [11].

**Table 3:** Effects of different carbon and nitrogen sources in diets on the growth performance of livestock and poultry

Model	Recommended CP level^a^, %	Crude protein, %	Crude protein reduction, percentage points^b^	Main source of starch	Main protein source	Amylose/Amylopectin ratio	Daily weight gain	Feed/gain ratio	N utilization	Reference
Pig (7.5 kg)	23.70	17.08 vs. 18.36^c^	5.34–6.62	Corn	Casein vs. corn gluten meal	——	Increase	Decrease	Increase	[97]
Pig (7.5 kg)	23.70	19.79 vs. 17.08	3.91–6.62	Corn	Casein	——	Increase	Increase	Increase	[97]
Pig (7.5 kg)	23.70	17.84 vs. 17.08	5.86–6.62	Corn	Casein vs. corn gluten meal	——	Increase	Increase	Increase	[97]
Pig (7.5 kg)	23.70	19.79 vs. 18.36	3.91–5.34	Corn	Casein vs. corn gluten meal	——	Increase	Increase	Increase	[97]
Pig (17.4 kg)	20.90	18.0 vs. 17.8	2.90–3.10	Corn	Soybean meal, DDGS vs. soybean meal	——	Decrease	Increase	NS^d^	[60]
Pig (17.4 kg)	20.90	17.9 vs. 17.8	3.00–3.10	Corn	Soybean meal, casein vs. soybean meal	——	NS	NS	NS	[60]
Pig (17.4 kg)	20.90	17.9 vs. 18.0	2.90–3.00	Corn	Soybean meal, casein vs. soybean meal, DDGS	——	Increase	Decrease	Increase	[60]
Pig (28.9 kg)	18.00	9.51 vs. 9.54	8.46–8.49	Waxy corn vs. waxy corn/high amylose corn starch	Casein	0.27	NS	——	NS	[11]
Pig (28.9 kg)	18.00	9.59 vs. 9.41	8.41–8.59	High amylose corn starch vs. waxy corn/high amylose corn starch	Casein	0.27	NS	——	NS	[11]
Pig (28.9 kg)	18.00	9.59 vs. 9.54	8.41–8.46	High amylose corn starch vs. waxy corn/high amylose corn starch	Casein	0.27	NS	——	NS	[11]
Pig (29.7 kg)	18.00	13.10 vs. 13.00	4.90–5.00	Waxy corn/corn starch vs. waxy corn	Soybean meal	0.20	NS	——	Decrease	[97]
Pig (29.7 kg)	18.00	13.31 vs. 13.00	4.69–5.00	Corn starch vs. waxy corn	Soybean meal	0.20	NS	——	NS	[97]
Pig (29.7 kg)	18.00	13.08 vs. 13.00	4.92–5.00	Corn starch/pea starch vs. waxy corn	Soybean meal	0.20	NS	——	NS	[97]
Pig (29.7 kg)	18.00	13.25 vs. 13.00	4.75–5.00	Pea starch vs. waxy corn	Soybean meal	0.20	NS	——	NS	[97]
Pig (29.7 kg)	18.00	13.31 vs. 13.10	4.69–4.90	Corn starch vs. waxy corn/corn starch	Soybean meal	0.20	NS	——	Increase	[97]
Pig (29.7 kg)	18.00	13.08 vs. 13.10	4.90–4.92	Corn starch/pea starch vs. waxy corn/corn starch	Soybean meal	0.20	NS	——	Increase	[97]
Pig (29.7 kg)	18.00	13.25 vs. 13.10	4.75–4.90	Pea starch vs. waxy corn/corn starch	Soybean meal	0.20	NS	——	Increase	[97]
Pig (29.7 kg)	18.00	13.08 vs. 13.31	4.69–4.92	Corn starch/pea starch vs. corn starch	Soybean meal	0.20	NS	——	NS	[97]
Pig (29.7 kg)	18.00	13.25 vs. 13.31	4.69–4.75	Pea starch vs. corn starch	Soybean meal	0.20	NS	——	NS	[97]
Pig (29.7 kg)	18.00	13.25 vs. 13.08	4.75–4.92	Pea starch vs. corn starch/pea starch	Soybean meal	0.20	NS	——	NS	[97]
Pig (32.3 kg)	18.00	13.90 vs. 13.40	4.10–4.60	Cassava vs. Corn	Soybean meal	0.20 vs.0.78	Increase	NS	NS	[49]
Pig (32.3 kg)	18.00	13.70 vs. 13.40	4.30–4.60	Corn, Cassava vs. Corn	Soybean meal	0.46 vs.0.78	NS	NS	NS	[49]
Pig (32.3 kg)	18.00	13.90 vs. 13.70	4.10–4.30	Cassava vs. cassava, corn	Soybean meal	0.20 vs.0.46	NS	NS	NS	[49]
Pig (43.3 kg)	18.00	15.69	2.31	Corn	Casein vs. corn gluten meal	——	——	——	Increase	[97]
Broiler (0–21 d)	23.00	21.00	2.00	Waxy corn vs. corn	Soybean meal	0.09	Increase	NS	——	[98]
Broiler (0–21 d)	23.00	21.00	2.00	Tapioca vs. corn	Soybean meal	0.09	NS	NS	——	[98]
Broiler (0–21 d)	23.00	21.00	2.00	Waxy corn tapioca	Soybean meal	0.09	Increase	NS	——	[98]
Broiler (0–24 d)	23.00	18.30 vs. 20.10	2.90–4.70	Corn	Soybean meal	——	NS	NS	——	[99]
Broiler (0–24 d)	23.00	19.60 vs. 20.10	2.90–3.40	Corn	Soybean meal	——	NS	NS	——	[99]
Broiler (0–24 d)	23.00	18.80 vs. 20.70	2.30–4.20	Corn	Pea	——	NS	NS	——	[99]
Broiler (0–24 d)	23.00	19.70 vs. 20.70	2.30–3.30	Corn	Pea	——	NS	NS	——	[99]
Broiler (0–24 d)	23.00	18.80	4.20	Corn	Pea vs. soybean meal	——	Increase	Decrease	——	[99]
Broiler (6–20 d)	23.00	18.50 vs. 21.0	2.00–4.50	Amylose	Soybean meal	——	NS	NS	——	[100]
Broiler (6–20 d)	23.00	18.50	4.50	Waxy rice vs. corn	Soybean meal	——	Increase	Decrease	——	[100]
Broiler (6–20 d)	23.00	18.50	4.50	Amylose vs. corn	Soybean meal	——	NS	Decrease	——	[100]
Broiler (6–20 d)	23.00	18.50	4.50	Waxy rice vs. amylose	Soybean meal	——	Increase	Decrease	——	[100]
Broiler (10–28 d)	20.00	14.00	6.00	Corn	5% DDGS vs. 12.5% DDGS	——	Decrease	Increase	——	[101]
Broiler (14–35 d)	20.00	17.50	2.50	Sorghum vs. wheat	Soybean meal	——	Increase	Decrease	——	[102]
Broiler (22–42 d)	20.00	18.24	1.76	Corn starch vs. cassava starch	Soybean meal	——	NS	——	——	[103]
Broiler (22–42 d)	20.00	18.24	1.76	Corn starch vs. pea starch	Soybean meal	——	Increase	——	——	[103]
Broiler (22–42 d)	20.00	18.24	1.76	Cassava starch vs. pea starch	Soybean meal	——	Increase	——	——	[103]
Goose (4–10 weeks)	15.00	11.50 vs.16.50	3.50	Corn	By-products vs. soybean meal	——	Increase	Decrease	——	[104]
Goose (4–10 weeks)	15.00	14.00 vs.16.50	1.00	Corn	By-products vs. soybean meal	——	Increase	Decrease	——	[104]
Goose (4–10 weeks)	15.00	11.50 vs.9.80	4.50–5.20	Corn	By-products	——	NS	NS	——	[104]

^a^ Values for pigs and poultry were calculated based on Nutrient Requirements of Swine (1998) and Nutrient Requirements of Poultry (1994), respectively

^b^ Crude protein reduction level was calculated as the difference between the recommended CP level and the dietary CP level

^c^ vs. denotes the contrast between the experimental and control groups with respect to crude protein level, feed ingredient, or amylose/amylopectin ratio

^d^ NS indicates no significant difference between groups (*P* > 0.05); —— indicates missing or undetermined values. All studies were conducted under low-protein diet conditions

### Improvement in meat quality

The dietary amylose to amylopectin ratio has an important effect on meat quality. A higher proportion of amylose is often associated with decreased fat deposition, whereas in some studies, a diet with a higher proportion of amylopectin increased intramuscular fat deposition, reduced the postmortem glycolysis rate, and improved the muscle pH, thereby enhancing meat quality. In terms of muscle tissue, a diet containing a low amylopectin/amylose ratio improved water-holding capacity and tenderness, reduced drip loss and meat firmness, and was associated with increased muscle fiber density, reduced fiber diameter, and increased MyHC-IIa expression alongside decreased MyHC-IIb expression in finishing pigs [111, 112]. These findings suggest that such a diet promotes the transition of muscle fibers toward a mixed oxidative/glycolytic phenotype, thereby improving meat quality. Concurrently, reducing the amylopectin/amylose ratio in the diet decreased lactate content in the longissimus dorsi muscle of finishing pigs, downregulated *HK-2* expression, and upregulated *ATP5B* expression [112]. These findings suggest a reduction in muscle glycolytic potential and an enhancement of mitochondrial oxidative energy production, which may be a key factor in improving post-slaughter muscle metabolism and meat quality.

Notably, the effects of starch with a high amylose/amylopectin ratio on muscle fiber type transformation in finishing pigs are not entirely consistent, and this variation may be related to the starch source. Pea starch, which has a high amylose/amylopectin ratio, exhibits unique effects on meat quality that differ from those of conventional starch structural regulation. It increases daily weight gain in finishing pigs, reduces the feed-to-meat ratio, increases eye muscle area, and reduces backfat thickness [113, 114]. Pea starch can also simultaneously decrease glycogen phosphorylase activity, glycogen content, and glycolytic potential, increase adenosine triphosphate (ATP) and adenosine diphosphate (ADP) levels, and decrease phosphocreatine (PCr) and adenosine monophosphate (AMP) levels [115]. This indicates that pea starch can improve meat quality by reshaping the preslaughter muscle energy metabolism state and reducing excessive post-slaughter glycolysis. A certain proportion of pea starch can enhance pork marbling, intramuscular fat, and water-holding capacity; reduce shear force; increase pH and myosin solubility; and promote the transition of muscle fiber type from the glycolytic MyHC-IIb to the oxidative MyHC-I and MyHC-IIa, which overall contribute to improved meat quality. At the post-transcriptional regulation level, the downregulation of miR-23a and upregulation of its target genes, as well as the upregulation of miR-499 and downregulation of its target genes in muscle, also suggest that pea starch may participate in muscle fiber remodeling regulation through miRNA regulation [113, 114].

Overall, the regulation of lipid metabolism and meat quality by the carbohydrate and protein adaptation exhibits distinct tissue specificity and feed dependency. A high amylose/amylopectin ratio tends to reduce blood and liver lipid levels, inhibit fat synthesis, and promote lean meat deposition, but may increase muscle firmness [116]. Starch with a high amylose/amylopectin ratio from specific sources, such as pea starch, can simultaneously optimize carcass traits and enhance meat quality [113–115].

### Regulation of intestinal health

Intake patterns of protein and carbohydrate are key factors driving changes in gut microbial diversity and community structure, as well as maintaining gut homeostasis [117]. An appropriate glucose and amino acid release pattern can regulate host health by influencing the gut microenvironment, increasing the proportion of carbohydrate fermentation, promoting SCFA production, and improving gut barrier homeostasis. Studies on pigs have shown that a diet with a low amylopectin/amylose ratio (0.14) significantly increases acetic acid and butyric acid levels in the ileocecal region and propionic acid levels in the cecum of finishing pigs while significantly enriching the phylum Bacteroidetes and the genus *Prevotella*, whereas a high amylopectin/amylose ratio (0.43) reduces certain *Gammaproteobacteria* and increases *Lactobacillus acetotolerans*-like bacteria [118]. In low-protein diets for weaned piglets, reducing the amylopectin/amylose ratio did not fully restore growth performance but reduced the incidence of diarrhea, increased fecal α-diversity, and enriched potential SCFA-producing bacterial groups, such as *Prevotella* and *Faecalibacterium* [119], while reducing harmful bacteria such as *Streptococcus* [11]. Studies on poultry have found that the regulatory effect of the carbon source structure on microbial communities is similarly pronounced. Starch with a low amylopectin/amylose ratio (0.20) can reduce the richness of the gut microbiota in broiler chickens [120]; in contrast, starch with a moderate amylopectin/amylose ratio (0.3–0.5) helps enrich potential beneficial bacterial communities in the cecum of laying hens, such as *Lactobacillus*, Rikenellaceae, and Christensenellaceae, and improves nitrogen utilization efficiency [121].

In addition to the amylopectin/amylose ratio, the starch source also significantly influences the gut microbiota. Although pea starch has a high amylose/amylopectin ratio, it increased the relative abundance of starch-degrading bacterial communities, such as *Lactobacillus* and *Streptococcus*, in the cecum of finishing pigs, while reducing pro-inflammatory bacterial communities (e.g., *Tyzzerella*, *Porphyromonas*, and *Tannerella*) and harmful metabolites (e.g., histamine and indole) [122]. This suggests that the starch source exerts an effect on microbial regulation that is independent of the amylopectin/amylose ratio. However, pea starch increased the dry matter flow of chyme in the terminal ileum of growing pigs, reduced the digestibility of various amino acids, and elevated protein fermentation products, such as isobutyric acid and isovaleric acid [106, 123]. This suggests that when carbon source release is relatively slow and out of synchronization with the supply of available nitrogen sources in the foregut, underutilized nitrogen is more likely to be transported to the hindgut and undergo protein putrefactive fermentation. Similarly, although cassava, as a source of amylopectin, can improve feed intake, weight gain, and nitrogen efficiency, it is also associated with increased diarrhea rates, foul-smelling gases in feces, and elevated levels of isobutyric and isovaleric acids [49]. Wali et al. [124] found, within the geometric framework for nutrition, that under low-protein diets, different carbohydrate sources can lead to significantly different metabolic fates. Compared with monosaccharide combinations that readily induce adverse metabolic responses, resistant starch, which is released more steadily during digestion and exhibits more favorable fermentation characteristics, is more beneficial to health.

Carbohydrate and protein adaptation is also associated with changes in digestive enzyme activity and intestinal structure. Broiler chickens that were fed a nitrogen-free diet exhibited intestinal atrophy and a decreased villus-to-crypt ratio, while mucus secretion increased, goblet cell numbers increased, and the *MUC2* gene was upregulated. As the amylopectin/amylose ratio increased, maltase activity in the intestine decreased linearly, while treatment with a moderate amylopectin/amylose ratio more closely resembled the normal digestive physiology [120]. This indicates that the matching between the structure of carbohydrate and nitrogen supply affects the digestive absorption and barrier maintenance capabilities of the intestinal epithelium.

### Increased intestinal nutrient transport

The use of a corn/barley composite starch diet significantly upregulated the expression of the glucose transporter *SGLT1* and the amino acid transporters *B*^*0*^*AT1*, *EAAC1*, and *CAT1* in the first segment of the small intestine (duodenum) of weaned piglets, thereby increasing the concentration of free amino acids and nitrogen deposition in the portal vein and improving nitrogen use efficiency [44]. Feeding weaned piglets a 14% crude protein diet significantly reduced the transcription levels of nutrient transporters (such as *CAT1* and *PepT1*) in the jejunal mucosa, whereas a low-protein diet supplemented with casein hydrolysate improved intestinal barrier function and regulated the expression of transport-related proteins [125, 126]. A low amylose/amylopectin ratio also upregulated the expression of the glucose transporters *GLUT2* and *SGLT1* in the porcine jejunum, suggesting that changes in carbon source structure can improve the efficiency of carbon–nitrogen synchronization by enhancing glucose absorption capacity in the small intestine. At the same time, decreased expression of *SMCT1* and *FFAR3* in the ileum indicated that hindgut fermentation and SCFA signaling may also have been altered [54]. However, a diet with a low amylose/amylopectin ratio also elevated fasting blood glucose and insulin levels, suggesting that when there is an excessive shift toward rapidly digestible carbon sources, the body’s glucose metabolism and hormonal secretion patterns also undergo adjustments [111].

In poultry, the impact of carbohydrate and protein adaptation on nutrient utilization efficiency is equally evident. A high amylopectin/amylose ratio significantly improved egg weight and the feed-to-egg ratio, and the amylopectin/amylose ratio showed a linear positive correlation with amino acid digestibility, metabolizable energy, and the villus area, indicating that an appropriate carbon source structure helps enhance the nutritional utilization efficiency of laying hens. Furthermore, high-amylose starch increased eggshell thickness and crude protein digestibility while reducing amino acid oxidation, suggesting that during the laying phase, slowly-releasing carbon sources may also improve nitrogen utilization efficiency under certain conditions [121]. In broilers, high-amylose starch reduced maltase activity and dry matter digestibility, while causing a linear decrease in endogenous amino acid losses, which suggests that changes in the digestion rate of carbohydrate sources can simultaneously affect digestive enzyme activity, nutrient digestion, and endogenous losses [120]. There is a significant interaction between the starch source and lysine levels in broiler chickens. Combining an appropriate starch source with higher levels of digestible lysine can increase body weight, improve the feed conversion ratio, and upregulate the expression of related genes such as *y*^+^*LAT1* and *mTOR*; the mechanism may be related to improvements in intestinal morphology, enhanced amino acid digestion and absorption, reduced amino acid oxidation for energy in the intestinal mucosa, and increased amino acids redirected toward muscle protein synthesis [98]. On the other hand, broiler chickens may have different needs for carbon source release patterns at various growth stages. Increasing the proportion of rapidly digestible starch in the diet during the early growth phase helped improve nutrient utilization and production performance, whereas appropriately increasing the proportion of slowly digestible starch in the later growth phase was more conducive to maintaining production performance, suggesting that carbon–nitrogen adaptation exhibits certain stage-specific characteristics [100].

### Regulating fat metabolism

**Fig. 4:**
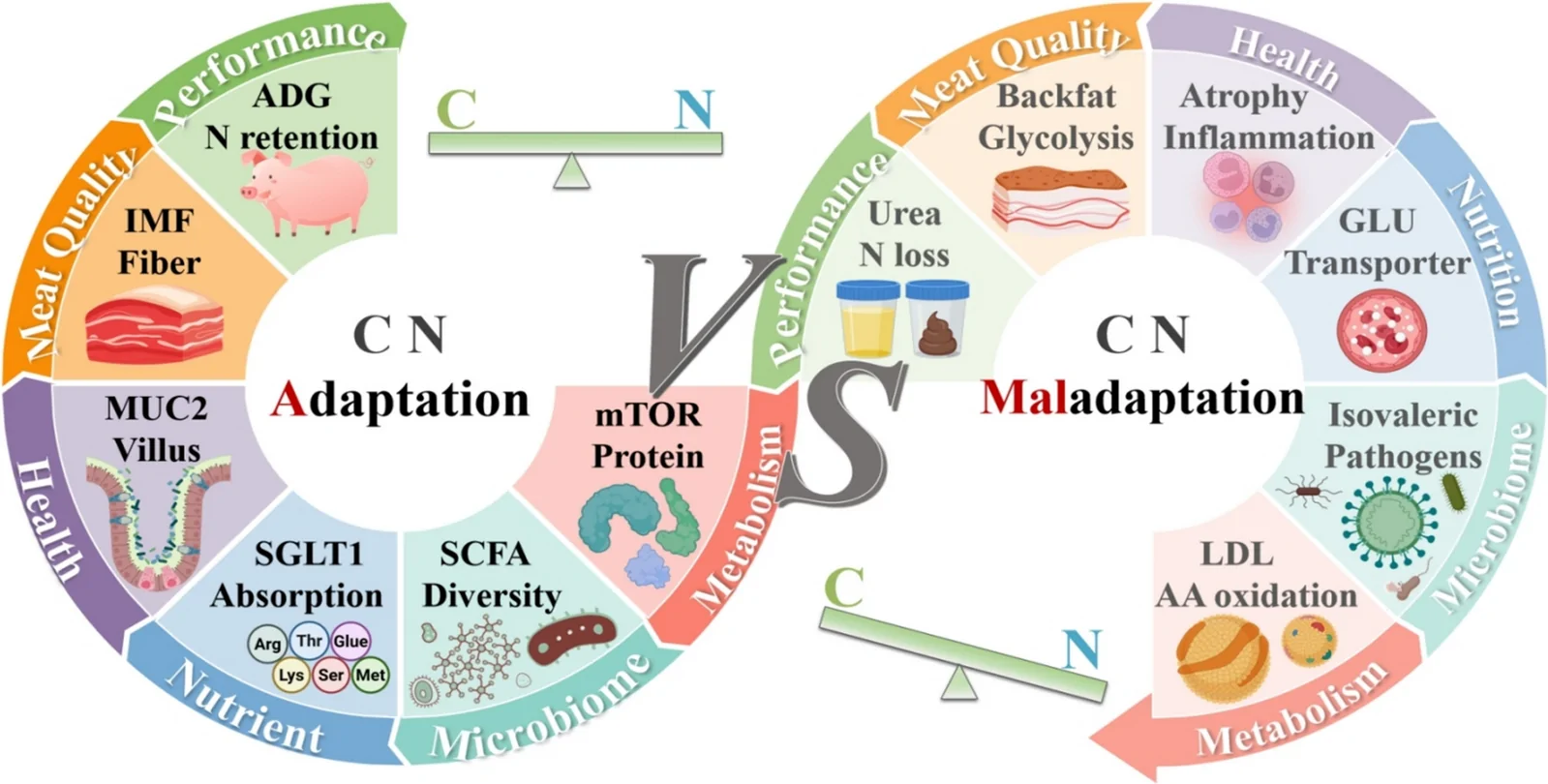
Effects of a low-protein diet based on carbohydrate and protein adaptation on the health of livestock and the underlying mechanism. The figure was created in BioRender (https://BioRender.com/dgtx4gg)

Studies on pigs have shown that pea starch increases docosahexaenoic acid (DHA), eicosapentaenoic acid (EPA), and n-3 polyunsaturated fatty acids (PUFA) levels, upregulates fat synthesis-related genes, such as *FAS*, *LPL*, *SCD*, and *ACCα*, and downregulates fatty acid oxidation-related genes such as *CPT1B*, accompanied by elevated levels of tetradecanoyl-L-carnitine and monoacylglycerol, indicating that pea starch significantly alters muscle lipid deposition patterns and fatty acid composition [114, 122]. Regarding signaling pathways, although insulin receptor expression decreased, *mTORC1* and *S6K1* expression increased, suggesting that high-amylose starch may reshape metabolic allocation in the liver and muscles by altering energy supply mechanisms and nutrient sensing signals [127]. A diet with a low amylopectin/amylose ratio increased total liver fat and cholesterol content and reduced serum low-density lipoprotein cholesterol (LDL-C), accompanied by decreased hepatic hexokinase and pyruvate kinase activity, as well as increased expression of *FoxO1* and *FAS*, suggesting enhanced gluconeogenesis and lipogenesis [54] (Fig. 4).

## Future research prospects

Current studies mostly focus on general definitions of “fast” and “slow” for the digestion and fermentation of ingredients and qualitative descriptions of “good” and “bad” for the synchronic release of dietary nutrients. Future research must achieve breakthroughs in the following areas:

### Extension of carbohydrate and protein adaptation research from monogastric animals to ruminants

To date, research on carbohydrate and protein adaptation has primarily focused on monogastric animals such as pigs and poultry. Although ruminal energy-nitrogen synchronization has been investigated in ruminants, systematic studies on post-ruminal (host-level) carbohydrate and protein adaptation in ruminants remain scarce. Given the distinctive rumen fermentation and the growing need to improve nitrogen utilization efficiency in addition to optimizing microbial protein synthesis, there is an urgent need for systematic investigations into host-level carbohydrate and protein adaptation in species such as cattle.

### Quantitative and precise characterization of glucose and amino acid release patterns

The majority of studies conducted to date have primarily focused on starch source, starch structure, digestibility, ingredient effects, or nutrient transporter expression. Due to the technical difficulties in precisely controlling dual release kinetics in vivo, direct experimental validation is currently lacking. Consequently, our current synthesis of evidence necessarily rests on inference and extrapolation from these indirect studies. To move beyond this limitation, the field must prioritize validation studies explicitly designed to examine carbohydrate and amino acid synchronization under precisely controlled in vivo conditions, with particular emphasis on the simultaneous regulation of carbon and nitrogen release rates. Achieving this will require an integrative approach that combines dynamic in vitro digestion simulations, real-time in vivo monitoring, and emerging platforms such as porcine organoids and organoid-on-a-chip, thereby deepening our understanding of the temporal kinetics of feed ingredient fermentation and digestion.

### In-depth molecular mechanism analysis of carbohydrate and protein adaptation using multi-omics approaches

By integrating multi-omics technologies, including transcriptomics, metabolomics, proteomics, microbiomics, and epigenomics, an in-depth analysis of the underlying molecular mechanisms by which carbohydrate and protein adaptation influences nutrient sensing signaling pathways (mTOR, AMPK), protein synthesis pathways, glucose and lipid metabolic networks, and gut microbiota-host interactions in livestock and poultry should be conducted. In addition, key molecular targets and regulatory networks underlying the carbohydrate and protein synchronized release, nitrogen deposition, muscle fiber remodeling, and the directed regulation of lipid metabolism should be identified, thereby providing a molecular biological foundation for the precise regulation of carbon–nitrogen adaptation and facilitating an innovative transition from phenotypic regulation to molecular design.

### Carbohydrate and protein adaptation modification and synergistic utilization of non-conventional feed raw materials

Taking into account the characteristics of China’s feed resources, the glucose and amino acid release characteristics of non-conventional feed raw materials, such as cottonseed meal, rapeseed meal, distillers’ grains, and fungal residues, should be investigated, and targeted processing and modification technologies (enzymatic hydrolysis, fermentation, and extrusion/puffing) to specifically regulate the carbon and nitrogen release rates should be developed, thereby aligning them with the glucose and amino acid rhythms of conventional feed ingredients. This can help overcome the bottleneck in protein feed resources and achieve the efficient utilization of non-conventional feed resources, which is a key innovative direction for the localization and industrialization of carbohydrate and protein adaptation technology.

## Conclusions

Carbohydrate and protein adaptation is a core innovative approach for overcoming technical bottlenecks in low-protein diets for livestock and poultry. It moves beyond traditional static energy and nitrogen balance theories to focus on the dynamic digestive and metabolic interactions between carbohydrates and proteins. By quantitative matching and gradient combination of the glucose and amino acid release characteristics of raw materials, carbohydrate and protein adaptation can achieve temporal synchronization, spatial alignment, and functional synergy of carbohydrate and protein nutrients, thereby fundamentally addressing the issue of low nitrogen utilization efficiency caused by energy and protein imbalance. In practical application, the innovative principles of dynamic matching, gradient combination, targeted regulation, and systemic adaptation must be followed to achieve the efficient synergistic utilization of carbohydrate and protein nutrients. In the future, research on carbohydrate and protein adaptation technology must advance toward precise quantification, deepened mechanistic understanding, diversified raw materials, and intelligent regulation. Furthermore, it should establish a comprehensive theoretical framework and technical solutions, promote the adaptation and utilization of unconventional feed ingredients and the development of cross-species common technologies, and facilitate the industrial implementation of carbohydrate and protein adaptation technology, thus providing core innovative technical support for the livestock industry to overcome protein feed resource bottlenecks and achieve nitrogen reduction, emissions reductions, and sustainable development.

## Data Availability

No datasets were generated or analysed during the current study.

## Abbreviations

ADP: Adenosine diphosphate
AMP: Adenosine monophosphate
ATP: Adenosine triphosphate
DDGS: Distillers dried grains with solubles
LDL-C: Low-density lipoprotein cholesterol
NSP: Non starch polysaccharides
PCr: Phosphocreatin
SCFA: Short chain fatty acid
